# Natural outbreaks and bioterrorism: How to deal with the two sides of the same coin?

**DOI:** 10.7189/jogh.10.020317

**Published:** 2020-12

**Authors:** Lionel Koch, Anne-Aurelie Lopes, Avelina Maiguy, Sophie Guillier, Laurent Guillier, Jean-Nicolas Tournier, Fabrice Biot

**Affiliations:** 1 Bacteriology Unit, French Armed Forces Biomedical Research Institute (IRBA), Bretigny sur Orge, France; 2 Pediatric Emergency Department, AP-HP, Robert Debre Hospital, Paris, Sorbonne University, France; 3 CBRNE Joint Defense Center (CIA NRBC), Saumur, France; 4 Risk Assessment Department, University of Paris-Est, French Agency for Food, Environmental and Occupational Health & Safety (ANSES), Maisons-Alfort, France; 5 Department of Microbiology and Infectious Diseases, French Armed Forces Biomedical Research Institute (IRBA), Bretigny sur Orge, France

For the last three decades, the outbreak events have constantly increased and became more complex to prevent, predict and contain. Nowadays, health authorities concern is to identify which ones are bioterrorist outbreaks. However, natural outbreaks and biological attacks have a too intertwined nature to be considered separately and hence, in the absence of any attack evidence, differentiate them is a delicate task requiring complex, long and rigorous scientific investigations. Furthermore, and as demonstrated by the COVID-19 outbreak, the effectiveness of the response to an outbreak is closely dependent on the timeliness of the response: the effort should thus rather focus on the development of early detection and preparation measures such as the development of global contingency plans organising the action of all entities (civilians, militaries, governmental and non-governmental) in a common effort. Innovative Artificial Intelligence is becoming unavoidable to detect the crisis and to manage it, especially in the phases of preparedness and response effectiveness. This technology largest impact will be to complement and enhance human capabilities but cannot substitute them. The human experts monitoring new threats and able to work with these systems will stay at the centre of the stage.

In the last thirty years, the pace of emerging infectious disease outbreaks has significantly increased [1]. The globalisation of international exchanges contributes to the inefficiency of common quarantine measures to contain the disease [2]. The last Ebola outbreak in 2014 in West Africa was regarded as a paradigm of the issues caused by emerging infectious diseases nowadays: this extremely deadly pathogen has naturally emerged in a large new area, and its overwhelming spread has subsequently impacted Europe and the United States [3]. This observation was confirmed and emphasised by the coronavirus disease pandemic (COVID-19) caused by the new human coronavirus SARS-CoV-2. The effectiveness of the ongoing lockdown of billions of people during the COVID-19 will have to be evaluated and compared to other strategies. Thus, outbreaks can no longer be considered as a local and distant issue but should be regarded as a global concern [4].

Of course, in History, some outbreaks have been the starting point of biological attacks, even long before the discovery of microbiology. In 1346, Mongols exploited the second plague pandemic by catapulting the bodies of soldiers died from plague over the city walls of Caffa [5]. In the same lines, in the 18th century, the distribution of infected blankets from a smallpox hospital of English settlers probably caused the deadly smallpox outbreaks in the Native Americans population [5]. In the 20th century, after the discovery of microbiology, a period of extensive industrial biological weapon programs started with scenarios of massive biological attacks against military troops. Since 2001, the threat is considered more focused on actions against the population or vital interest points of the nations. These biological attacks could be perpetrated by state or non-state groups in the context of low intensity or hybrid wars and bioterrorist attacks [6].

Nowadays, when an outbreak occurs, one of the first concern of the authorities is to separate a natural outbreak [7] from an intentional act involving a biothreat agent [5] in order to adapt their management. Even the SARS-CoV-2 did not escape the suspicion to have been laboratory-engineered [8,9].

Thus, this review will show that there are no easy ways to distinguish one from each other but that they share the same consequences and hence should have a shared management. Accordingly, group together preparation measures and response tools against both the emergence of an unknown pathogen and an unpredictable attack will optimise the effectiveness of the response.

## NATURAL OR INDUCED OUTBREAK: HOW TO DISTINGUISH THEM?

The Biological Weapons Convention signed in 1972 outlaws the use of biological weapons [10]. Since then, the identification of a biological attack is a major international political and judiciary issue [11]. However, multiple nested events such as global warming [12], natural catastrophes [13], human actions [14] and conflicts [15] affect natural outbreaks in an unpredictable way [16]. Several authors proposed algorithms to determine, during crisis or shortly after, if the biological event had natural or induced causes [17-19]. Except for some criteria, like evidence of a release explicitly referring to attacks, the great part of the arguments should be carefully analysed before being attributed to a biological attack.

### The agent specificity

The use of some spontaneously rare agents could denote a criminal origin, as it has been the case with the use of *Bacillus anthracis* during the Amerithrax crisis in 2001 [20], and, to a lesser extent, with the Aum Shinrikyo sect in Japan in 1993 [21]. However, the agent is not a sufficient criterion to identify natural or induced biohazard. For example, the Rajneesh sect used a quite common *Salmonella enteric* [22] to perpetrate attacks, and some bacterial toxins are considered as a potential warfare agent precisely because of their high prevalence [23]. In sharp contrast, recent natural outbreaks involved top select agents like Ebola virus in West Africa in 2014-2015 [3] or *Yersinia pestis* causing pulmonary plague in Madagascar in 2017 [24]. Even the emergence of a peculiar new strain cannot be a stand-alone criterion to differentiate both events. Indeed, even if there is no evidence of using such agents through history, natural agents can be modified by humans to increase their transmission, lethality or drug resistance capabilities [5]. At the same time, some natural outbreaks were caused by naturally altered pathogens like the *Escherichia coli* O104:H4 in Europe in 2011, a strain that acquired and combined unusual virulence factor and drug resistance genes [25] or in 2003 the new human coronavirus (SARS-CoV) identified with surprise in front of severe acute respiratory syndrome cases [26].

**Figure:**
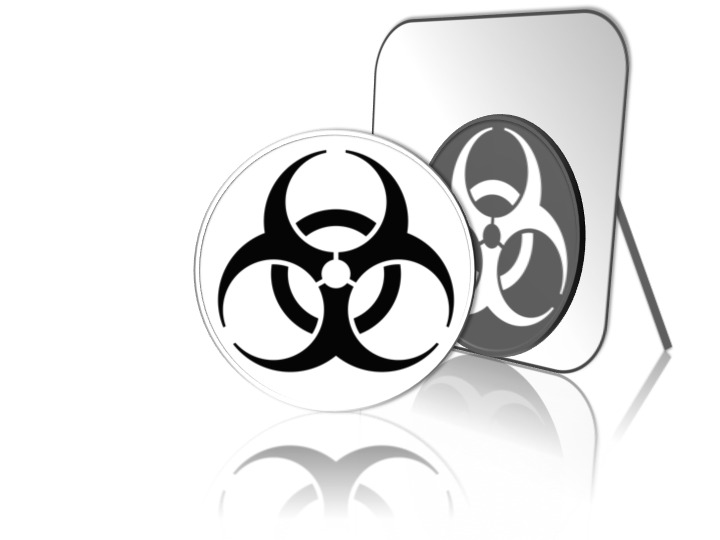
Photo: Two sides of the same coin (from Lionel Koch’s collection, used with permission).

### The spatial and temporal distribution

If a pathogen is detected in a location where it has never been detected before, it can constitute a hint for a biological attack suspicion. It was the case with the Amerithrax crisis in 2001 when a Texan *B. anthracis* strain was found on the East Coast of the USA [20]. However, the biggest outbreak of the Ebola virus occurred in a part of the continent recognised as free of the disease until then [27]. One other clue for biological attacks could be the seasonality, arguing that if an outbreak appears during a season not compatible with the pathogen time-life, human activity could be the cause [5]. Here too, some natural outbreaks disrupted all rules like the Influenza virus H1N1 pandemic in 2009, which appeared in April in North America with two epidemiological spikes [28]. It unusually emerged from infected pig populations and was followed by a unique global spread [29].

### The origins and dynamics

Multiple starting points are commonly considered a sign of a biological attack like the five letters containing *B. anthracis* spores [20] as well as the several restaurants targeted by the Rajneesh cult [22]. An attack can also occur in a single place, like the “*Shigella dysenteriae* poisoning” in a laboratory in 1996 in the US, where one unique set of pastries has been deliberately contaminated by a laboratory strain [30]. In contrast, the natural tularaemia outbreak in Kosovo in 1999-2000 reached several districts simultaneously in a tensed geopolitical context [31] and, in 2017, the plague outbreak in Madagascar had multiple index cases [24]. Even the assumption that an unusual swift spread or a large share of the population rapidly affected could be evidence for a biological attack is disputable: recent terrorist actions used non-contagious pathogen and hence reliable epidemiological data for the intentional use of a contagious disease do not exist [5]. By contrast, the influenza virus [28], the 2003 SARS-CoV [26] and the SARS-CoV-2 [32] propagated very fast all around the world with more than 200 countries affected in one year for the first one and 30 countries in 5 months for the second. For the current COVID-19 pandemic, the Centre for Systems Science and Engineering (CSSE) of Johns Hopkins University (Baltimore, MD, USA) created a website to visualise and track the reported cases in real-time [33]. In April 2020, less than five months after the first alert, 185 countries declared at least one case of infection (https://coronavirus.jhu.edu/map.html). In the same vein, the last Zika virus natural outbreak showed an unusual spread, as it emerged in Africa, travelled across the Pacific Ocean to finally trigger a pandemic in South America [34].

### Is there any interest to identify one from the other?

Thus, to characterise an infectious phenomenon, we should merge the most advanced technics with thorough epidemiological investigations. Results have to be interpreted very carefully by taking into account contextual elements and technical biases to avoid any misunderstanding [35]. The list of common-sense items is beneficial to process data and improve awareness but should not be solely used to distinguish the origin of an ongoing event because of a lack of reliability (**Table 1**). It should be noted that all criteria and weightings have been determined retrospectively based on past outbreaks and bioterrorist attacks. One of these algorithms has been reviewed in the light of new infectious events but have not yet proven its effectiveness on a prospective basis [36]. The confusion surrounding these criteria confirms that both phenomena have intertwined nature. Moreover, during a natural outbreak, depending on the knowledge about its hazardousness and transmission, the infectious agent can be secondarily regarded as a biothreat agent, like it is now the case with the US department of justice currently considering people who intentionally spread the SARS-CoV-2 as terrorists [37]. However, these political considerations are far away from health workers and do not consider the public health issues of quick detection and response. Indeed, even if the substantial remaining risk in the case of an attack is the possibility of secondary actions aiming to maximise damages to the emergency infrastructure [38], the real challenge for global safety remains the early detection, the accurate characterisation and the establishment of specific measures, whatever the outbreak origin [39,40]. During the COVID-19 crisis, it had been estimated that the early detection and isolation of cases would have been more efficient to prevent infections than travel restrictions and contact reductions [41].

**Table 1:** Published consensual criteria to assess an unusual outbreak and infectious events in function of the presence or the absence of criteria

Criteria*	Present	Absent
**Selected agent**	Amerithrax (USA)†	Rajneesh attack (USA)†
	Aum Shinrikyo attack (Japan)†	Common bacterial toxins†
	Neurotoxin botulinium A (Worldwide)‡	*Shigella dysenteriae* (USA)†
	Ebola virus (West Africa)‡	
	*Yersinia pestis* (Madagascar)‡	
**Emergence or altered pathogen**	*E coli* O104:H4 (Europe)‡	All biological attacks†
	SARS-CoV (Worldwide)‡	
**Unusual distribution**	Amerithrax (USA)†	
	Ebola outbreak (West Africa)‡	
	H1N1 Influenza (Worldwide)‡	
**Multiple starting points**	Amerithrax (USA)†	*Shigella dysenteriae* (USA)†
	Rajneesh attack (USA)†	Aum Shinrikyo attack (Japan)†
	*Francisella tularensis* (Kosovo)‡	
	*Yersinia pestis* (Madagascar)‡	
**Unusual spreading**	H1N1 Influenza (Worldwide)‡	All biological attacks†
	Coronavirus (Worldwide)‡	
	Zika virus (Worldwide)‡	

*Consensual criteria.

†Induced cause.

‡Natural cause.

## HOW TO EARLY DETECT THE UNEXPECTED?

### The challenge of an early detection

Some diseases like influenza are internationally monitored [42] while some others are subject to active surveillance in an outbreak context, like the Ebola virus during the last outbreak in West Africa [43]. For such well-known diseases, the case definition is clear and an outbreak is declared when the number of cases exceeds what has been expected [44]. This classic and passive way of detection is efficient but has numerous drawbacks as it requires an expensive and complex public health network and is often activated with a certain delay. However, when it comes to a new disease or pathologies with polymorphic or nonspecific symptoms, the case definition and the outbreak declaration threshold are subject to debate [45]. The source of the infection can be as unpredictable as the local outbreak of anthrax in reindeers triggered by a permafrost melting [46] or the detection of the variola virus in ancient mummies [47].

Most of the time, the high volatility lying in the infectious process complicates the record of the cases. For the same exposition, symptoms can differ according to individual variables like health status or genetic factors [48] or to collective variables involved, for example, in the chain of transmission [49] but also cultural or socio-economic factors: the most-disadvantaged individuals will develop more severe and hence more specific forms of the disease but will have a belated use of health care [50].

On the other hand, systematic environment monitoring for all diseases is, for now, impossible due to technological barriers and cost challenges. Experts in biodefense suggested that more targeted measurements in delimited spaces or during a large gathering of people should be the priority to improve the sensitivity and specificity of the early detection of a biological attack but, also for a natural outbreak, while reducing the cost [51]. For example, the analysis of wastewater could be a good way to monitor the spread of SARS-CoV-2 in the community [52]. However, these measurements should always be paired with epidemiological investigations to avoid any misinterpretation of the results [51].

Thus, for the moment, health workers would first notice an unusual event (disease or an unusual number of cases) and should be able to alert public health officials [44] as protecting themselves from contagiousness. Given the importance of early detection, training has to be a building block in infectious diseases programs in order to promote unusual event awareness [53]. The implementation of information technologies leaves room for improvement in the outbreak detection process [54] as more and more stakeholders of the health care system use informatics tools in their daily practice. Yet, considerable efforts have been made on information technologies and electronic query of a data set to improve the efficiency of surveillance [55]. It's an imperative prerequisite for the implementation of an electronically assisted surveillance. Currently, data management tools can aggregate several inputs and are already used for epidemiological studies or trigger an alert [56].

### To a connected age

Internet-based surveillance systems offer a logistically and economically appealing extension to this traditional surveillance approach. The results are immediate and allow access to a paucisymptomatic population or people who are not using the health care system [57]. This methodology has been used in 2020 in China to reconstruct the progression of the SARS-CoV-2 outbreak [58]. Despite ethical concerns and regulatory barriers, social networks appear to be a powerful data collection tool and can also be used as a medium to communicate sanitary messages or alerts [59]. However, here again, these data are subject to many biases and should be carefully interpreted. Indeed, the simple act of online documentation is just an indirect marker of disease, and such detection system could trigger an alert just because a worldwide released blockbuster movie increases the anxiety of population or a massive hacking produces millions of requests.

Taken to its logical extreme, the next step of this epidemiologic watch would probably allow the contribution of the internet of things (IoT) already used to follow chronic illness [60] and for biomedical research [61]. A smartphone or a smartwatch is now able to detect modifications of vital parameters like temperature or heart rate. The capability of crossing these types of information with, for example, geo-tracking solutions, could alert competent authorities on an ongoing infection and help them to implement more rapidly appropriate measures and focus on a possible source of contamination. This seems to be only the beginning of IoT possibilities as the future could be even more connected with the development of projects like smart cities. Nowadays, China is already using video surveillance systems to follow its citizens and detect incivilities [62]. Likewise, criminality hot spots prediction by artificial intelligence (AI) is no more fictional in Los Angeles [63]. These new technologies already have some applications in epidemiology as the detection in real-time of restaurants with a higher risk to be sources of foodborne diseases [64]. In the medical field, computers start to help clinicians in the diagnostic of mental illness through the facial expressions and head gesture in a video [65] but could also be used to detect an infectious disease at the prodromal phase with potential highest efficiency than thermic portals. The crossing data obtained from surveillance systems combined with machine learning capabilities in prediction and diagnostic could be used to help early detection of an infectious phenomenon in a population. This early detection could guide further specific actions of screening to identify potential patients and even the source of the infection. In Korea, during the COVID-19 crisis, GPS from cellular phone, credit card transaction log and video footage had been used to monitor the patient’s contact and avoid further transmissions [66]. However, the implementation of such surveillance systems is not without legal and ethical issues and should be carefully considered. The privacy policy has to be carefully examined as well as the securing of the transmission and storage of sensitive medical data, not to mention the possible human rights abuses in non-democratic countries [67]. These concerns have already been raised during the current COVID-19 pandemic [68] but there is still no international consensus on the use of personal data.

**Figure 1:**
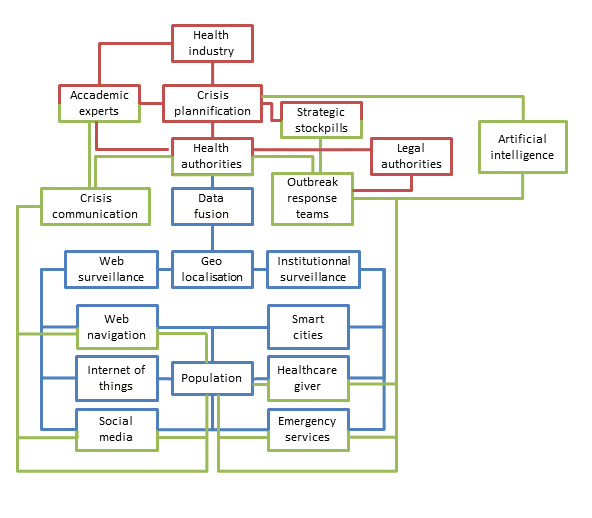
Possible future detection and management system for outbreaks. In blue: the population and the detection resources for infectious events. In red: the stakeholders of the crisis preparation and management. In green: the response to the outbreak through communication and specific actions.

Pending the advent of AI tools, many initiatives have been recently proposed to facilitate the investigation of epidemics in the genomic era. The whole-genome sequencing already can help to determine the origin of an outbreak and also to explain the dispersion of the pathogen through its local evolution [69]. New tools may include online data processing [70] up to the development of original algorithms to aggregate spatial, temporal, epidemiological and genomic data [71]. The interactions of this technological surveillance system with the previously described classic one are also possible at the condition to continue to improve the data-sharing practices [72]. The use of the Nextstrain tool [73] in the context of SARS-CoV-2 (https://nextstrain.org/ncov) perfectly illustrates the potential of such approaches in the context of spreading epidemics [74]. In the years to come, the epidemiological monitoring system of our societies will probably rely on economic capacities, technical development capabilities and societal choices, with the common objective to early detect outbreaks, regardless of their causes (**Figure 1**).

## CRISIS MANAGEMENT

### Early detection for an early reaction

Even if the epidemiological monitoring is the crucial step to respond to an outbreak, detection is useless if the resources to deal with the crisis are unavailable. Being prepared includes but is not limited to health workers being trained to detect, react and alert the health authorities: quick and reliable equipment has to be available and health workers have to be used to work with them. Dedicated infrastructures have to be prepared and ready for activation and Personal Protective Equipment (PPE), intensive care devices and treatments have to be stockpiled. The COVID-19 crisis revealed that the lack of simple PPE put the all health system at high risk [75]. Several authorities (civilians or militaries, governmental or non-governmental entities) already have some contingency plans but the compartmentalisation between different governmental branches and the nebulous labelling of the means between natural outbreak or bioterrorist attack dedicated sometimes prevent an accurate global appreciation [76]. As it is, and as unfortunately still demonstrated during the COVID-19 pandemic, if an outbreak would occur, there is a risk, even for the highly trained first aid service in the most developed countries, to get in each other’s way. By learning how to work together, synergies could be developed to improve health response [77]. After the failure in the control of the last Ebola virus outbreak by the WHO, international agencies called for better international preparation to respond to future outbreaks [78]. Thus, international and European research networks managed to improve the speed and effectiveness of the present deployment on a validated diagnostic workflow for SARS-CoV-2 [79]. This demonstrating the response capacity that can be released through the coordinated action of academic and public laboratories like PREPARE [80]. In 2020, in China, coordination by the central authority allowed to deploy medical staff and built new hospitals in Wuhan in a tight schedule. In Europe, crisis management strategies were different among countries, but cooperation helped relieve overloaded Intensive Care Units in some regions and saved lots of patients. In the meantime, other consortiums like GRACE may also help us to prepare the possible future sanitary crisis [81].

### Preparedness technologies

Developments of AI do not only help for early detection, but make available a full set of possibilities in crisis management to the authorities. By using classic risk analysis documentation with AI tools, it is now possible to generate predictions to improve the resilience of a system and to mitigate the risk [82]. The preparation phase of the crisis can also benefit from AI tools by ordering the reuse of information from previous crises [83], improving the stockholders’ training with a serious game approach [84], helping to design realistic plans [85] or even boosting the discovery of new drugs [86]. Resources management can also be improved by the use of AI in terms of network structuration [87] as well as for the mean’s allocation [88]. During the crisis, AI can also sort information from many sensors, merge it and make it relevant for the user responsible of the situation assessment [89], which will be helped by a decision-support system [90] to design the best crisis response. For example, during the COVID-19 crisis, social contact matrices had been used to project the benefit to maintain social distancing measures [91]. Over the past ten years, epidemiological and mathematical modelling data were essential for risk characterisation and management during infectious disease outbreaks [92] but ironically, the rising power of AI systems will not erase the role of human experts [93]. Indeed, intuition and emotions are known for a long time to be part of the decision-making process [94]. During crisis management, expert intuition developed through years of practice is described as more realistic than pure analytical thinking [95]. Moreover, both intuition and creativity are part of the problem-solving process [96]. Both experts and AI will have to learn how to work together and assist each other by developing collaborative intelligence, which will be the best way to solve complex problems (**Figure 1**). This was experienced during the COVID-19 crisis in which experts, assisted by simulations, had to make decisions to control the spread of a virus still little known.

## DIMENSIONING THE GLOBAL PREPAREDNESS

### Economic impact

Inevitably, to develop an anticipation-centred view required investment. The economic justification of such an investment was underlined for a long time (even before the Amerithrax crisis) [97], and recently, a panel of USA experts recommended reinforcing the biological threat characterisation research at a federal level with clear safety, ethical and practical guidelines [98]. Splitting outbreaks into two causes is not cost-efficient and seems absurd as dangerous pathogens to human can be used for biological attacks but are first and foremost causing natural outbreaks [99]. However, studies about the burden-adjusted research intensity showed that diseases with a greater impact are still underfunded [100] in an economical context where citizens are more and more concerned by public expenses. Indeed, if the vaccine policy implemented were economically profitable in the USA during the 2009 Influenza pandemic [101], a similar strategy caused substantial wastage in Australia [102]. Thus, authorities have to be very careful to dimension their actions appropriately, even though a delayed response is severely judged by public opinion as during the 2014 Ebola outbreak [78]. Hence, authorities and experts should improve the global contingency plans, especially on catastrophic biological risks, which represent a small portion of the biological threats but with substantial potential consequence for humanity [103].

For a health care system, the preparation for a biological attack [6,104] or a natural outbreak [78,105] is globally the same challenge. Moreover, preparedness for biological attacks has a significant added value that helps to strengthen preparedness for natural outbreaks, and vice versa [104]. It is therefore economically interesting to consider the natural biological risk and the possibility of an attack as a single threat in the preparation of the response to an infectious event with epidemic potential. The crisis generated by the numerous deaths of COVID-19 and the lockdown of billions of people will probably trigger a new evaluation of public policies to control outbreaks with the opportunity that the public opinion will look at it through fresh eyes.

### Misguidances and consequences

Indeed, the uncertainty associated with scientific knowledge often challenges decision making and opens the way to the contestation of expertise [92]. Sometimes, the best intentions can result in a health disaster, such as the deployment of a peacekeeping force and the cholera outbreak in Haiti in 2010 [106] or the project of spreading some modified mosquitoes to fight against malaria [107]. Technology allows us to modify organisms specifically leading to the reconstitution of the Spanish Influenza virus [108] or to unexpected results as a test for a new *poxvirus* vaccine resulted with an ultra-virulent strain able to neutralise the immune system [109] or, during research experiments mimicking natural phenomenon, the generation of highly-resistant *B. anthracis* spores [110] and viruses acquiring airborne transmission [111]. Nowadays, these widely used technics appear to be almost obsolete in comparison with the new capacities of gene synthesis: a *horsepox virus* has been reconstructed using only internet-bought sequences [112], and a new bacterium has been created de novo in a laboratory [113]. Currently, the possibilities of genome editing technologies like CRISPR-Cas9 seems to be limitless [114]. Some malicious scenarios have already been imagined with a genetically modified virus infecting mosquitoes able to perform gene modification of crops in a field [115]. The South African « coast » project [116] that aimed at developing a bacterial agent able to selectively kill a part of the population could now be a terrifying technical possibility. Thus, even if applications of some of these modified organisms may be highly beneficial, as the recycling complex wastes [117], they are swamped in the middle of the wanderings reported by the media [118].

Due to all these miscalculations and misguidances, society lost confidence in the authorities and national programs. It leads to society-born threats with notably the growing emergence of highly antibiotic-resistant bacteria due to the improper antibiotic use [119] or the re-emergence of nearly forgotten pathogens linked to the mistrust in public health programs like vaccination programs [120]. This lack of confidence extends to crisis management programs and can compromise outbreak management measures the same way it happened with the Ebola outbreak in 2014 [121] or currently, with the beginning of the management of the COVID-19 pandemic and the lockdown decision [122]. However, during the COVID-19 pandemic, the transparency about its progress reported in real-time, for the first time in the outbreaks’ history, lead to better comprehension and cooperation of people [123]. Thus, every decision can have a dual nature and should be considered carefully before being implemented (**Table 2**). That is why, while encouraging research, technologies and their application must be controlled to avoid any misuse and major communication actions are needed to overcome the public reluctance. Ethics in research and data publication must also be placed at the centre of researchers' concerns.

**Table 2:** Duality of decisions in infectious phenomenon management.

Type of change	Positive effect	New risk
Science progress	Better understanding of infectious process	Creation of new threats
Internet screening	Weak signal detection	Data manipulation
Open data	Sharing of the knowledge	Misuses of the data
Improved surveillance systems	Early detection and characterisation	Privacy and human right violation
Use of AI	Collection and fusion data	Lose of human expertise
Increased communications	Better acceptance from the population	Fake news

AI – artificial intelligence

## CONCLUSION

The intricate nature of natural outbreaks and biological attacks is too important to consider them separately. To create an efficient way to detect and contain them, the first step is to anticipate them by performing continuous scientific and epidemiological monitoring. Still, the most serious and unpredictable events are referred as “Black swan events” and despite our inability to foresee their occurrence, knowledge keeps being the key concept to anticipate them [124]. Thus, we need to continue and intensify networking at local, regional and global levels. Stakeholders from a broader range of backgrounds must be involved to monitor the evolution of threats and update existing procedures by developing concrete and immediately applicable solutions in crisis. The biological crisis is becoming a field of expertise by itself, and it is no longer enough to be a specialist in crisis management, infectious disease or epidemiology to be able to understand the implications of their own decisions fully. New technologies and AI will have more impact on crisis management, and experts will have to better work with these tools to improve their preparedness. The evolution of threats as well as technologies developments will require permanent adjustments in the strategies to optimise the public health response. Communication will also be a key point of the future strategies to promote the acceptance of financial and societal investment by both the public authorities and the population and to avoid false information spreading. Current COVID-19 crisis is the first pandemic to benefit from so much advanced research and several major articles are published every day. However, SARS-CoV-2 is probably not the deadliest virus we will ever have to fight. We must learn from this crisis while preparing the next one.
